# Role of Gasotransmitters in Oxidative Stresses, Neuroinflammation, and Neuronal Repair

**DOI:** 10.1155/2017/1689341

**Published:** 2017-03-12

**Authors:** Ulfuara Shefa, Seung Geun Yeo, Min-Sik Kim, In Ok Song, Junyang Jung, Na Young Jeong, Youngbuhm Huh

**Affiliations:** ^1^Department of Biomedical Science, Graduate School, Kyung Hee University, 26 Kyungheedae-ro, Dongdaemun-gu, Seoul 02447, Republic of Korea; ^2^East-West Medical Research Institute, Kyung Hee University, 26 Kyungheedae-ro, Dongdaemun-gu, Seoul 02447, Republic of Korea; ^3^Department of Otorhinolaryngology, H & N Surgery, College of Medicine, Kyung Hee University, 26 Kyungheedae-ro, Dongdaemun-gu, Seoul 02447, Republic of Korea; ^4^Department of Applied Chemistry, College of Applied Science, Kyung Hee University, Deogyeong-daero, Giheung-gu, Yongin-si, Gyeonggi-do 17104, Republic of Korea; ^5^Department of Reproductive Endocrinology and Infertility and Department of Obstetrics and Gynecology, Cheil General Hospital, Dankook University College of Medicine, 17 Seoae-ro 1 Gil, Jung-gu, Seoul 04619, Republic of Korea; ^6^Department of Anatomy and Neurobiology, College of Medicine, Kyung Hee University, 26 Kyungheedae-ro, Dongdaemun-gu, Seoul 02447, Republic of Korea; ^7^Department of Anatomy and Cell Biology, College of Medicine, Dong-A University, 32 Daesingongwon-ro, Seo-gu, Busan 49201, Republic of Korea

## Abstract

To date, three main gasotransmitters, that is, hydrogen sulfide (H_2_S), carbon monoxide (CO), and nitric oxide (NO), have been discovered to play major bodily physiological roles. These gasotransmitters have multiple functional roles in the body including physiologic and pathologic functions with respect to the cellular or tissue quantities of these gases. Gasotransmitters were originally known to have only detrimental and noxious effects in the body but that notion has much changed with years; vast studies demonstrated that these gasotransmitters are precisely involved in the normal physiological functioning of the body. From neuromodulation, oxidative stress subjugation, and cardiovascular tone regulation to immunomodulation, these gases perform critical roles, which, should they deviate from the norm, can trigger the genesis of a number of neurodegenerative diseases such as Alzheimer's disease (AD) and Parkinson's disease (PD). The purpose of this review is to discuss at great length physical and chemical properties and physiological actions of H_2_S, NO, and CO as well as shedding light on recently researched molecular targets. We particularly put emphasis on the roles in neuronal inflammation and neurodegeneration and neuronal repair.

## 1. Introduction

Gasotransmitters are endogenously synthesized gaseous molecules with vast physiological modalities in the human body. Gasotransmitters are produced and highly regulated by enzyme systems and their functioning is not dependent on a particular receptor [1]. Three main gasotransmitters have been identified: nitric oxide (NO), carbon monoxide (CO), and hydrogen sulfide (H_2_S). Evidence shows that these gasotransmitters are involved in the origin of life and have roles in the endosymbiotic events that contribute to the biogenesis and development of mitochondria [2]. Oxidative stress is the production of reactive oxygen species (ROS) in amounts exceeding the ability of the body's antioxidant systems to counteract their effects [3]. These free radical species which contain one or more unpaired electrons act as electron donors, causing oxidation which leads to the potential damage to body macromolecular polymers such as lipids, proteins, and nucleic acids [4]. Perhaps most important of all cell targets of ROS are nervous system cells, especially neurons which are highly susceptible to the harmful effects of ROS [5]. Neuronal cells have relatively primitive antioxidant defense systems rendering these cells prone to oxidative modifications. Though glial cells (microglia and astrocytes) are the pivotal support cells of the nervous system, they are the crux of most neuroinflammatory processes. Neuronal cells' close association with these glial cells renders neurons vulnerable to the ROS and acts as effective pathogenic elements in initiating many neurodegenerative diseases like Parkinson's disease (PD), Alzheimer's disease (AD), amyotrophic lateral sclerosis (ALS), HIV-1-associated dementia (HAD), and so forth. Free radicals have the ability to invade proteins, polysaccharides, lipid bilayers, and DNA which cause oxidative damage to cells. Oxidation in nucleic acid causes disease conditions which is detected by increasing levels of 8-hydroxyl-2-deoxyguanosine in DNA and 8-hydroxyguanosine in RNA. Hydroxyl radical causes DNA damage which results in DNA strand breakage and DNA protein cross-linking as well as base modifications. The total set of events lead to neuronal injury [6].

**Figure 1:**
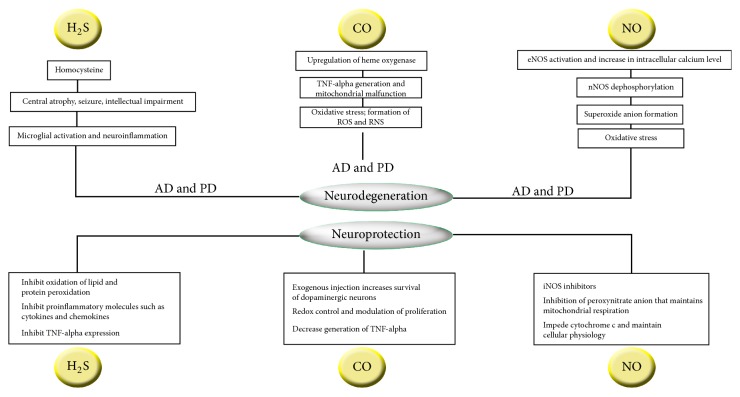
Neuroprotective and neurodegenerative aspects of gasotransmitters (H_2_S, CO, and NO) including the pathways of their roles in development of Alzheimer's disease (AD) and Parkinson's disease (PD). Hydrogen sulfide (H_2_S) causes neurodegenerative diseases (AD and PD) by its precursor homocysteine which causes endothelial dysfunction and vascular diseases as well as inflammation on macrophages, microglia, astrocytes which result in central atrophy, seizure, and intellectual impairment and H_2_S gives neuroprotection by inhibition of lipid and protein peroxidation as well as cytokines, chemokines, and TNF-*α* (tumor necrosis factor-*α*) expression. Carbon monoxide (CO) causes neurodegeneration by upregulating heme oxygenase eventually mitochondrial dysfunction and reactive species formation such as ROS (reactive oxygen species) and RNS (reactive nitrogen species) but recent discovery shows that it gives neuroprotection by controlling redox formation and reducing production of TNF-*α*. Nitric oxide (NO) causes neurodegeneration by activating eNOS (endothelial NO synthase) which increases intracellular calcium (Ca^2+^) level following nNOS (neuronal NO synthase) dephosphorylation and oxidative stress but, by using iNOS (inducible NO synthase) inhibitors, inhibition of peroxynitrite anion, and halting cytochrome c, it maintains homeostasis.

H_2_S protects nerves from oxidative stress, shields photoreceptor cells in the retina of the eye from degeneration of the photo induced effects, monitors endoplasmic reticulum damage, and protects kidney from ischemia/reperfusion injury. Neuromodulation and neuroprotection are the common features of H_2_S and are effective in many pathological conditions like age related diseases [7]. NO can act as both neuroprotective and neurotoxic free radical (Figure 1). For neuroprotection, it uses several mechanisms like Akt kinase and cyclic-AMP-responsive-element binding (CREB) protein pathways [8, 9]. NO can be toxic if it is produced in excess [10]. Moreover, NO is much more harmful under pathological conditions which results in the production of ROS; for example, its interaction with superoxide anions results in the formation of peroxynitrites [10, 11]. In a study conducted by Cousar et al., it is shown that there is an increased level of HO-1 which is the CO producing enzyme after severe traumatic brain injury in the CSF of infants and children [12]. Hence, HO-1 protein is also a significant serum biomarker for initial assessment of AD because this protein increases in patients with AD and also mild cognitive impairment [13]. Also, HO-2 which is constitutively expressed in the mammalian brain and testis is another CO producing enzyme and its expression has been found to increase in instances that involve cell damaging stimuli [14, 15] such as hypoxic ischemic insult [14]. Additionally, CO influences astrocyte-neuron communication in a paracrine fashion as a form of neuroprotective mechanism [16].

The purpose of this review is to describe how gasotransmitters act on central nervous system (CNS), their pathophysiological role in oxidative stress associated neurodegenerative diseases, and these gases' modulation of neuroinflammation. We also delineate the possible molecular targets targeted by these gases and emphasize their therapeutic potential.

## 2. Gasotransmitters (NO, H_**2**_S, and CO)

Given that these gaseous molecules are endogenic, they are poised to have a great deal of biological impact on the physiological systems of the body, especially with regard to the relative quantities within a particular tissue at a given moment in time [17, 18].

### 2.1. NO

Of the three gases, the first to be fully described and its roles elucidated is NO. Its synthesis is catalyzed by NO synthase (NOS), an enzyme that exists in three isoforms. NO production in the mammalian body is a result of an enzymatic reaction involving L-arginase as the substrate. In normal physiologic conditions, NO is tightly regulated, with its fundamental role well recognized in vasodilation [19]. NO is remarkably known as the endothelium-derived relaxing factor (EDRF) [20]. First described by Furchgott, he reported that some components like acetylcholine can stimulate the endothelium to generate volatile substances that could directly induce relaxation of the smooth muscles of blood vessels, resulting in naming it EDRF. This molecule would later be recognized as NO [16, 21, 22]. Other researchers showed that the same molecule was able to activate macrophages, hence facilitating effective phagocytic destruction of tumor cells and bacteria. Slowly it is becoming clear that NO function starts to spread broadly with the separation and molecular cloning of neuronal NO syntheses (nNOS or NOS1), which subsequently causes cloning of macrophages to form endothelial NO synthase (eNOS) and inducible NO synthase (iNOS) [23]. NO biosynthesis involves the conversion of guanidine nitrogen of l-arginine to l-citrulline. NOS enzymes are regulated by FAD (flavin adenine dinucleotide), FMN (flavin mononucleotide), and tetrahydrobiopterin and phosphorylated by a various kinds of serine kinases. Rapid stimulation of eNOS and nNOS effect is caused by calcium-calmodulin innervation and the translocation between the intracellular alveolar structure and the plasma membrane [24].

NO also prohibits adherence of platelet and leukocyte's adhesion to endothelium, which is the downregulating process that downregulates the proinflammatory events [25, 26]. Evidence shows that when aging occurs, it causes reduction in the release of NO because of endothelial cell dysfunction [27]. Many pieces of evidence show that the dysfunction of endothelium is a typical feature of aging [28, 29]. Vascular pathologies such as hypertension, hyperglycemia, or hyperlipidemia and chronic cerebral hyperfusion which are age related diseases are caused by profuse discharge of NO through eNOS activation [30]. Hence, chronic eNOS stimulation might result in endothelial cells dysfunction, decrease NO release from the endothelium, and decrease ability to perform normal vascular perfusion, causes blockage which adjoins the granulocyte in the walls of blood vessels, and thus halts proinflammatory reactions. This incidence increases fattening of basement membranes in common capillaries of the brains in Alzheimer's disease (AD) patients [31, 32]. This continuous discharge in microvascular regions causes decreased supply of glucose and oxygen; thus the neuronal and glial cells die [33]. NO inflation is caused either by excessive expression of NOSs or by any other system like excitotoxicity of glutamate. In addition, it causes the increase of intracellular calcium that increases nNOS dephosphorization and its enzymatic activity. NO interacts with the superoxide anion formation while metabolism of dopamine, thus peroxynitrite is considered as the one of the pivotal catastrophic molecules in the dopaminergic neuronal cells [34].

### 2.2. CO

CO is a highly poisonous, odorless, colorless, and tasteless gas. It is very inflammable in air over a wide range of concentrations and burns in air with a bright blue flame. It becomes a liquid at 81.62 K (−191.53°C) and is insoluble in water above 70°C [35]. In many studies it was identified that CO acts as an endogenous biological messenger in the brain and an essential element in the modulation of cerebrovascular circulation in neonates [36]. However, in our body, CO is produced via heme metabolism by heme oxygenase-1 (HO-1) and heme oxygenase-2 (HO-2) [37]. Heme oxygenase-1 (HO-1) is an inducible enzyme, whereas heme oxygenase-2 (HO-2) is constitutively expressed; these isoforms both are engaged in CO production. By tightly binding to iron atoms and hemoglobin, they efficiently curtail the oxygen bearing capability of those proteins; hence inhalation of high doses for long periods of time can cause tissue hypoxia [38]. Heme proteins are the main precursors for the origination and transduction of signals and intercommunication with CO, NO, and H_2_S neurobiology. The proteins have specific functions like transportation of genes, transport of electrons, cooperation of the oxidation-reduction reactions which take place at the catalytic sites of distinct enzymes, and sensing of different genes. CO balances production of adenosine triphosphate (ATP), metabolism of glucose, and energy that eventually maintains cellular respiration [39]. An in vitro experiment shows analysis with the help of using purified enzymes to coordinate the structure of heme binding pockets with the assistance of catalytic reactions which helps to complete sensing of genes and transduction of genes and their mechanisms [40–53]. Interaction of heme oxygenase/CO axis is thought to be a therapeutic target for different kinds of neurodegenerative diseases. Degraded product of heme is catalyzed by heme oxygenase; thus CO is regarded as a great neuroprotective agent [54–59]. In an experiment named bicuculline model of seizures with piglets, Parfenova and Colleagues showed that HO-1 inducer stabbed seizure activity [60].

A receptor, P2Y13, mediates the stimulation of the Nrf2/HO-1 axis that leads to neuroprotection. Nrf2 stimulation induces HO-1, which is inevitable in cellular defense against oxidative stress [61, 62]. Human immunodeficiency virus (HIV) diseased brain downturns extrusion of HO-1 which is completed with discharge of toxic levels of glutamate to neurons. So, HO-1 administration can be a therapeutic strategy for neuroprotection in case of HIV infection [63].

CO is proven to be neuroprotective in many animal models in the case of brain injury [64–68]. Safety and tolerability studies showed inhalation of CO in the case of the grown-ups and in newborns. Inhalation of CO has been newly introduced in the clinical trials and is considered to be an important therapeutic option. Although previous studies had shown the negative aspects of CO in the brain, recent studies revealed that the drug could be a pivotal therapy for inmates and could be used for healing of neuropathologies, psychiatric diseases, and neurodegeneration. Clinical trials uncovered that CO is needed for recovering from diseases of the patients. CO as well as nitric oxide may be considered as innovative treatment approaches for CNS diseases and conservation of health.

CO preserves the cell against generation of ROS and enormous production of NO. Administering CO exogenously could be an essential protective way of rebuilding the nervous system and changing the metabolism of neurons but the effectiveness is heavily dependent on the dose and the time of inhalation. Different reviewers showed that CO hinders apoptosis, raises the Bcl-2 expression, enhances the level of ATP, modulates the oxidative metabolism, curtails the production of lactate, diminishes glucose consumption, and elevates the action of cytochrome c oxidase in neuronal physiology [69, 70].

### 2.3. H_2_S

H_2_S is the third gasotransmitter which was recently discovered after NO and CO. It has a characteristic smell of rotten eggs and is produced in the mammalian cells through the enzymatic reactions of cystathionine *γ*-lyase (CSE), cystathionine *β*-synthase (CBS), and 3-mercaptopyruvate sulfurtransferase (3MST). L-cysteine and homocysteine as well as their derivatives are the typical substrates of the H_2_S production reaction [71]. H_2_S is considered an essential signaling functional molecule fragment as it has many regulating functions in the physiological system such as neurotransmission and neuromodulation and it is also associated with learning, memory, and nociception [72–74].

Chemically, H_2_S is a toxic gas and it has poisonous effects in almost all organs in our body and it functions in the body as a gaseous signaling molecule and it is involved in many physiological processes responsible for pathogenesis of many kinds of neurodegenerative diseases, diabetes, and heart failure [75, 76]. By interpreting the internal levels of H_2_S, beneficiary effects in the mammalian tissues could be discovered [77]. H_2_S has a similar structure to water and has very thin intermolecular force and is colorless and odorless [78]. It has P^H^ of 7.4 in the mammalian body [79]. Many animal studies show that H_2_S exists in quantities as much as 50–160 *μ*mol/L in tissues and its physiological donor is sodium hydrogen sulfide (NaHS) [71]. H_2_S is also considered as the endothelium-derived hyperpolarizing factor (EDHF) [80, 81].

The three gasotransmitters have different physical and chemical properties and they contribute differently by modulating body functions.

### 2.4. Salient Features about Gasotransmitters

Research on NO was started in the early 1990s but the research on CO and H_2_S has just started. During the first 10 years (2004–2013), scientists were able to find many applications and vital physiological relevance of gasotransmitters in the body. Day by day, the H_2_S research and the interest in NO and CO are increasing. New types of programs such as the Gasotransmitter Research and Training (GREAT) program have been initiated recently in Canada as a part of the undergraduate and graduate curriculum in the universities. And most recent is the establishment of gasotransmitters report in 2012 (http://gaso-transmitters.eu/) in the European Network.

The gasotransmitters have significant functions in the human body. By knowing their properties and specific functions, we can easily understand their mechanisms and metabolisms in different parts of the body. Research is running broadly to know more about their movements and regulation in the body; we hope that slowly we can engage them for beneficial roles in different bodily impairments.

## 3. Evidence of Causing Neuroinflammation of Gasotransmitters (NO, CO, and H_**2**_S)

The causes of neuroinflammation in some aging and other metabolic diseases such as hypertension, diabetes, depression, dementia, and stroke [82] and these are the main contributors to neuroinflammation [83]. Local and systemic CNS inflammation in the, affects cerebral small vessel diseases (SVD) which is known as vascular dementia [84, 85] and results in chronic hyperfusion, resulting consequently in continuous death of oligodendrocytes; thus myelinated fibers are degenerated which is considered as the reason of increasing low-grade inflammation amplification of the risk of stroke [86].

Excessive production of NO causes generation of RNS, particularly ONOO^−^, which is considered as the most toxic derivative product of NO. The derivative also causes nitrosative stress in the neurons by affecting mitochondrial functions and many proteins which are associated with physiological functions of neurons [87]. NO is also responsible for inhibition of the iron-containing ribonucleotide reductase that generates deoxy ribonucleotides from ribonucleotides [88, 89]. By preventing the production of this enzyme, NO hinders DNA synthesis as well as cell division and it destructs DNA by nitrosylative as well as deaminative reactions that cause DNA breaks [90]. NO, produced by iNOS, is a major component of HIV-gp41 neurotoxicity [91] and spinal cord injury [92]. A study showed that NO production that is iNOS-mediated is stimulated by both IFN-*γ* and bacterial LPS or dsRNA that decreases the expression of myelin-specific genes that includes myelin basic protein (MBP) such as 2′, 3′-cyclic nucleotide 3′-phosphodiesterase, myelin oligodendrocyte glycoprotein and phospholipid in human primary oligodendrocytes that leads to death of oligodendrocytes [93].

HO/CO system is involved in many devastating neurodegenerative diseases such as AD, PD, and ALS. Overexpression of HO-1 is found in the brains of AD patients which is linked with neurofibrillary tangles [55, 94, 95] and senile plaques as well as fibrillary acidic protein-positive astrocytes. HO-1 expression is also increased in AD neocortex and cerebral vessels [96]. This HO-1 upregulation in AD is induced by inflated free heme which is associated with neurodegeneration and it also gives compensatory reactions that alter the damaging heme to the antioxidants biliverdin and bilirubin [97]. But the sequence of time between HO-1 expression and AD is still unclear.

Production of H_2_S in the nervous system is strictly regulated because overproduction of H_2_S has many detrimental effects, while inflammatory cytokines like interleukin-1*β* (IL-1*β*) mediated memory loss by stimulating expression of CBS that produces H_2_S. Furthermore, H_2_S has different signaling processes in the brain; for example, dysregulation of its metabolism is liable for neurodegeneration and also abnormal signaling of H_2_S is observed in case of AD and PD [98]. Another study demonstrated that deficiency in the endogenous generation of H_2_S to 1-methyl-4-phenylpyridinium causes iron-induced neurotoxicity [99]. Other studies demonstrated that an opposite of both increased levels of homocysteine and decreased levels of H_2_S is observed in the brains of AD patients. The imbalance of proportion of endogenous production of H_2_S is associated with homocysteine-induced neurotoxicity or neuroinflammation [100]. But, to the best of our knowledge, there is no information on potential role of H_2_S production which is Hcy-mediated of neuronal cell death. Homocysteine causes ROS formation which stimulates neuroinflammation [101].

So, from above discussion, it can be said that upregulation of iNOS and HO-1 causes neural damage in both cases of NO and CO, but, in case of H_2_S, it causes neuroinflammation because of imbalanced generation. But the actual mechanism through which they can cause neurodegeneration is still in doubt.

## 4. Oxidative Stress and Gasotransmitters

### 4.1. Gasotransmitters and Oxidative Stress in PD

#### 4.1.1. H_2_S, Oxidative Stress, and PD

The two major enzymes responsible for producing H_2_S from the cysteine are the cystathionine *γ*-lyase (CSE) and cystathionine *β*-lyase (CBS). Currently, mitochondrial 3-mercaptopyruvate sulfurtransferase (3MST) is revealed to produce H_2_S in both the brain and vasculature tissues. Cysteine aminotransferase (CAT) generates 3-mercaptopyruvate which generates 3MST, from which H_2_S evolves [102]. Parkinson's disease is one of the common neurodegenerative diseases which has various kinds of manifestations including cognitive deficiency and most prominently dementia that is characterized by the progressive loss of dopaminergic neurons in the substantia nigra (SN) [103].

Studies show that H_2_S provides protection to neurons against oxidative stress. Neuroprotective effects of H_2_S are found in glutamate induced death with the enhancement of cysteine and concentration of *γ*-glutamylcysteine later on that increases the GSH concentrations [78, 104, 105]. Y. Kimura and H. Kimura showed that H_2_S gives protection primarily by triggering glutathione (GSH) levels [106]. Other mechanisms involved are the following: H_2_S protects the immortalized hippocampal HT22 cells and thus activates the ATP sensitive K^+^ channels (K^+^-ATP channel). In neurons, K^+^-ATP channels are activated by oxidation; thus H_2_S is activated in the vasculature which is an evidence of regulation of physiology of H_2_S of blood pressure [107] and thus is aroused by a direct alteration of the proteins of the channel by H_2_S, which is called sulfhydration [108]. In this way, gasotransmitters are proposed to modify K^+^-ATP channels of the cultured neurons; they give protection against stress. In this way, vasodilation causes the stimulation of K^+^-ATP channels in the vesicles and this stops vascular damage which gives protection against neurodegenerative diseases. H_2_S also modulates BK channels. Telezhkin et al. [109] showed that recombinant BK channels and those which are expressed by type I cells of the carotid body are hindered by H_2_S which ultimately affects the gas to excite as an impaired chemoreceptor [110]. It is found that H_2_S can elevate kinase 1/2 (ERK1/2) activity in the extracellular receptor in the smooth muscle cells of the vesicles [111, 112]. In this way, H_2_S provides protection by regulating this channel in the central neurons. H_2_S impedes consumption of oxygen and that causes 6-OHDA invoked oxidation in nicotinamide adenine dinucleotide phosphate (NADPH); as a result, microglial cells in the midbrain are switched on which consecutively accelerate the accretion of proinflammatory factors in the subcortical regions of the forebrain [113]. It is the fundamental mechanism through which H_2_S downregulates neuroinflammation and degeneration. In a rat model of PD, 6-hydroxydopamine (6-OHDA) is induced and there was significant level of decrease of endogenous H_2_S in the SNs. It demonstrates that treating with H_2_S prevents accretion of proinflammatory factors in the striatum, 6-OHDA-evoked activation of NADPH oxidase, consumption of oxygen, and activation of microglia in the SN [103].

The neuroprotective act of H_2_S has also been described in other experimental rat models of PD, which is neurotoxin-induced [114]. By stimulating or repressing of various protein kinases, for example, PKC, PI3/Akt, P38, JNK, and the ERK-MAPKs [115], H_2_S downregulates oxidative stress, suppresses infection, and maintains antiapoptotic effects. As L-Dopa is a generally used drug for the remedy of PD and it has capacity to uphold dopamine levels, it cannot halt the advancement of PD. Furthermore, long-term treatment with L-Dopa may cause neurodegeneration and dyskinesia [116] by itself. H_2_S activates transporters of glutamate and its functioning causes sulfhydration straight through the ERK/MAPK pathway, which weakens the generation of ROS and reduces oxidative stress [103]. So, it demonstrates that combination of L-Dopa and H_2_S will be more efficient for the cure of PD. An experiment model with mouse was designed with 1-methyl-4-phenyl-1,2,3,6-tetrahydropyridine (MPTP) and probenecid given intravenously which results in the deterioration of dopaminergic neurons and is defensive against the toxicity which is MPTP-induced [117].

In this way, H_2_S not only has protective effects on peripheral tissues but also significantly treats damage of neurons in PD. Interestingly, a study shows that drinking coffee and inhaling cigarettes can forbid monoamine oxidase (MAO) [118], which indicates that there is a low possibility of the occurrence of PD in those individuals. Cakmak showed that coffee consists of* Prevotella*-evolved H_2_S and in cigarette smokers' bodies H_2_S is a commonly found component. H_2_S also contributes to weakening vascular dementia injury through preventing apoptosis by maintaining Bcl-2 and Bax expression [119]. However, excessive endogenous production and metabolism of H_2_S cause oxidative stress which is responsible for pathogenesis of neurodegenerative diseases including Parkinson's disease (PD) [120].

So, from the above discussion, we can see that the gasotransmitter H_2_S is defensive against formation of PD by altering different channels in the human body as they fight against oxidative stresses within the body and the misconception about H_2_S from the previous studies are became decline.

#### 4.1.2. NO, Oxidative Stress, and PD

CNS is vulnerable to oxidative stress because of its higher consumption of O_2_. In humans, although the brain possesses only a small percent of weight of the body weight, it occupies 20% of basal O_2_ consumption. Oxygen is needed everywhere in the brain; for example, neuron utilizes much of O_2_ for its growth through mitochondria and ATP also needed to retain low gradients (high intracellular K^+^, low Na^+^, and very low and free Ca^2+^). For the production of glucose, the brain utilizes about 4 × 10^21^ molecules of O_2_ per minute. In aerobes, mitochondria synthesize ATP which could be why neuronal cell death is caused by the deep hypoglycemia and with inhibitors of the ATP syntheses like rotenone or cyanide [34].

Respiratory chain of the mitochondria is liable for creating most of the ROS and remarkably the firstly produced superoxide anion (O_2_^∙−^) in human tissues. 1-2% of O_2_ is consumed generally converted to ROS in normal physiology. The other reason is that RNS from the nitric oxide (NO^*∙*^). This free radical of gases is a vital biological messenger and highly diffusible that exerts an essential role in the CNS physiology. There are three isoforms regarding NO^*∙*^ production: neuronal NO synthase (nNOS, type I), inducible synthase of NO (iNOS, type II), and endothelial synthase of NO (eNOS, type III), which generates huge amounts of RNS by the activated status of microglia (macrophages). In CNS, nNOS expression is modulated by both physiological and pathological responses involving most activity of NO [9]. NO promptly reacts with O_2_^∙−^ to generate peroxynitrite (ONOO^−^) which is considered as most reactively active RNS. Thus oxidative stress is caused by ROS and RNS in the nervous system. In disease conditions, they are proliferated by an extravagant amount notably NO^*∙*^, which is the resultant of stimulated microglia (iNOS) or endothelial cells (eNOS).

In almost all cases, PD pathogenicity is sporadic and research on the postmortem brain shows that ROS and free radical stress have an important role in causing PD [121]. Studies on postmortem PD brains and treatment with 1-methyl-4-phenyl-1,2,3,6-tetrahydropyridine- (MPTP-) treated mice demonstrate that NO has prominent role in causing PD. Death of nigral dopaminergic neurons is caused by MPTP in parkinsonian patients and in primates and rodents [122]. In glial cells, MPTP is altered to MPP^+^ and it is taken up by dopamine transporters of the dopaminergic neurons. Cell death is caused by the accretion of MPP^+^ which can inhibit the complex I of electron transport chain in mitochondria, which results in DNA damage and activates PARP-1 which is lethal to cell [123]. An experiment shows that administering NOS inhibitor (7-nitroindazole) in mice or mice lacking either nNOS or iNOS genes are more resistant to MPP^+^-induced neurotoxicity [124]. Another explanation is that nitration of *α*-synuclein abolishes its capacity to form fibrils. Hence, in an unmodified state, when coincubated, nitrated *α*-synuclein promotes formation of fibrils and accelerates the generation of the Lewy body like lesions. In this way, nitration of *α*-synuclein may increase the risk of Lewy bodies' formation in PD patients, but it is still unclear [125].

Multiple evidences demonstrate that NO is related to the damage of DNA, modification of proteins, and cytotoxicity; those are the common mechanisms involved in Parkinson's disease (PD) and neurodegeneration. Excessive expression of dopaminergic 5H-SY5Y neuroblastoma cells is more prone to apoptosis through nNOS exposed by the PD inducing drug rotenone. DNA damage is provoked by reactive nitrogen species (RNS) and by elevating generation of the neurotoxic species like peroxidation products (such as 4-4-hydroxynonenal) and hydrogen peroxide. RNS also prohibit ribonucleotide synthesis of DNA and cause over-single-strand DNA breakages [126]. Damage of DNA stimulates the secondary upregulation of tumor suppressor p^53^ genes and raises of nuclear enzyme named poly ADP-ribose polymerase (PARP-1), which causes apoptosis in PD animal models [124]. NO also causes alterations of proteins like nitration and nitrosylation. When nitration of protein occurs, it aids a nitro (-NO_2_) group into one of the two carbons in 3 positions of aromatic ring of tyrosine residues to produce nitrotyrosine.

Many reports show that NO acts as potent neuroprotective agent and it has antioxidant action in the brain of the experimental Parkinson's disease model [127–129]. Moreover, NO is shown to prevent peroxidation of lipid and lipoprotein of low density [130, 131] to shield against dopaminergic neurotoxicity which is neurotoxin-induced [132] and to protect cells from oxidative species in vivo by both antioxidative and antiapoptotic mechanisms [128]. NO promotes cellular transduction mechanisms, maintains plasticity of neurons [133], and represses apoptotic cell death of neurons [8]. In this way, NO shows neuroprotective and restorative actions after stroke [134, 135], after traumatic brain injury in Alzheimer's disease [136], and in states of depression [137].

So, it can be said that NO creates oxidative stress reacting with O_2_ which is directly involved in neurodegeneration but it has also role in the prevention of PD formation in our body.

#### 4.1.3. CO, Oxidative Stress, and PD

Evidence shows that upregulation of heme oxygenase-1 (where HO-1 is an inducible enzyme that has a capacity to produce CO from the destruction of heme) within the CNS imparts stability against oxidative as well as other stresses [138]. In both neurons and glia, upregulation of HO-1 happens because of these insults [69]. This kind of upregulation may be the reason of advancement of creating many neurodegenerative diseases like AD and PD, but its role is quite controversial. However, a study by Schipper et al. showed that introduction of HO-1 in the astroglial cells is harmful and it bolsters mitochondrial malfunction and finally oxidative stress [139]. Excessive expression of *α*-synuclein in neuronal cells enhances mitochondrial dysfunction which creates oxidative stress [140]. Another evidence showed that increased expression of neural heme oxygenase-1 causes oxidative damage of proteins in PD [97, 141].

Study with the postmortem human brain, in the substantia nigra in both PD and control specimens, it shows that expression of HO-1 in the substantia nigra in case of PD is greater than the control specimens [142]. Despite the accumulation of HO-1 in the development of PD, HO-1 is also associated with protection of neurons. In an experiment with rat model of MPP^+^- (1-methyl-4-phenylpyridinium-) induced PD, it was shown that exogenous injection of adenovirus containing human HO-1 gene enhanced the survival rate of dopaminergic neurons (DNs) and decreased the generation of TNF-*α* [143]. MPP^+^-induced nigral dopaminergic neuronal death is prevented by fibroblast growth factor IX through the upregulation of HO-1. In the mutation of PINK1 G309D, in the autosomal recessive form of PD, it was shown that there was difficulty in the generation of HO-1 in response to oxidative stress [144]. Although HO has both cytoprotective and neuroprotective actions, some researchers investigated that it induces neurotoxicity and it can be a therapeutic target for long-lasting neurodegenerative diseases such as PD [145]. In this case, the role of ferritin, one kind of protein, is important. It shows two isoforms, L and H, distributed all over the tissues. It has double role of protecting cells from the oxidative stress. As L-ferritin has iron nucleation properties, an alteration in the chain causes deposition of iron in the cerebellum, basal ganglia, and motor cortex, which causes autosomal dominant inheritance disorder (neuroferritinopathy) [146]. In contrast, Thompson et al. 2003 showed that, in a mouse model for PD, a deficiency of H-ferritin had a protective role against the harmful role of iron in neurodegenerative diseases. An important discovery shows that existence of the ferritin in mitochondria is expressed only in the brain and testis [81]. It shows that this mitochondrial ferritin has a neuroprotective role in case of AD and PD [147].

So it can be said that regular production of CO has contribution to maintaining body physiology as well as contribution to modulating PD formation but it can be neurotoxic and it causes oxidative stress when it is produced excessively.

### 4.2. Gasotransmitters and Oxidative Stress in AD

#### 4.2.1. H_2_S, Oxidative Stress, and AD

AD is also a common form of dementia which is pathologically indicated by the aggregation of senile plaques which contains stimulated microglia and amyloid beta (A-beta) peptides [148]. Cystathionine *β*-synthase (CBS) might be the primary source of H_2_S in the brain. As H_2_S is predominantly produced endogenously in brain from cysteine by CBS, lower levels of H_2_S in the brain of the AD patients are a prominent risk factor for advancement of AD [149, 150]. In 1996, CBS activation named S-adenylyl methionine was revealed which efficiently decreased in individuals with AD [31] because when endogenous level of H_2_S decreases, it causes accretion of homocysteine level in the brain eventually [151]. Many studies including in vitro and in vivo studies shows that H_2_S has a prominent role in cell growth promotion and preservation of the function of the factors like amyloid beta (A*β*) peptides, malondialdehyde (MDA), hypochlorite (HOCL), and 4-hydroxy-2-nonenal (4-HNE) which causes oxidative stress [15, 152]. In a study of adult male Wister rats, null H_2_S toxicity has the capacity to increase cognition by lowering A*β* plaques and triggering the APP, PST, and ON/4R-tau isoforms. In addition, NaHS, which is a H_2_S donor, inhibits oxidation of proteins and peroxidation of lipids in the cells of neuroblastoma of the patients with AD [153].

In the brains of the AD patients, cerebral atrophy, seizures, and intellectual disabilities are shown, which are caused by the autooxidation of homocysteine [154, 155], and hyperhomocysteinemia is also found in the brains of the AD patients [156]. In a rat model, it is shown that H_2_S has a protective effect and it diminishes oxidative stress and homocysteine-induced toxicity by its antioxidant properties in the adrenal medulla and smooth muscle cells of the vesicles [51]. In the pathogenesis of AD, synaptic dysfunction and vascular inflammation also play a critical role [157]. Currently, mRNA expression and synaptic proteins in C57B2/6 male mice studies clearly show that homocysteine induced plasma alteration combined with synaptic remodeling in the hippocampus [158]. In this way, H_2_S affects synaptic remodeling. Hence, the neurodegenerative diseases like AD and PD do not primarily evolve inflammation but various studies shows that the inflammation on the macrophages, microglia, and astrocytes contributes to the advancement of the two diseases [159]. It is concluded that H_2_S gives protection from A-beta-induced cell injury by preventing inflammation, increasing cell growth, and restoring functions of mitochondria [106, 148]. Thus, H_2_S preserves neurons from oxidative damage and degeneration in AD patients. By activating *γ*-GCS and cysteine transportation, H_2_S gives protection to neurons from glutamate mediated oxidative stress which accumulates excessive glutathione [106]. On the other hand, neurotoxicity arises by increasing levels of Hcy which is associated with the inhibition of the endogenous H_2_S synthesis; it could be a possible therapeutic strategy in AD which is Hcy-induced [100].

This paragraph gives an idea that H_2_S could be a possible therapeutic strategy in the treatment of neurodegenerative disorders.

#### 4.2.2. CO, Oxidative Stress, and AD

HO is a kind of enzyme which is broadly distributed within the body. It is also a cytoprotective enzyme which has some homeostatic and neuroprotective role in case of AD which is somehow controversial [147]. In CNS, within neurons and astrocytes, HO-2 is constitutively expressed but HO-1 is regarded as the inducible form in both cell types [55, 76, 142, 160]. By degradation of both HO-1 and HO-2, it liberates carbon monoxide (CO), biliverdin, and ferrous ion (Fe^2+^). This catabolism is essential for the metabolism of iron and bile which generates highly efficient antioxidants in bilirubin. Many stimuli impart the expression of HO-1 gene [161], which involves oxidative stress [162] and A*β* peptides [163]. As, AD is occurred by the elevated level of redox-active iron, constant exposure of oxidative stress and mitochondrial impairment which all are associated with pathological phenomena. Overexpression of glial HO-1 is noticed in experimental models which causes damage of mitochondrial membrane by oxidative stress and autophagy in astrocytes [164]. Moreover, long period of the excessive expression of HO-1 causes toxic tau accumulation in the mouse brain [165] and inflation of deposition of glial iron [166]. Interestingly, in AD patients, HO-1 is swiftly upregulated which is demonstrated as a marker of the oxidative stress [139, 142, 167]. In contrast, expression of HO is also noticed to decrease oxidative stress in an aged group of canine model that promotes cognitive dysfunction and neuropathology which is also similar to those that in human AD patients, increased levels of HO which is atorvastatin induced and it is also associated to reduce oxidative stress [168]. Α1-antitrypsin, which is a HO suppressor factor, is liable for generation of AD and an elevated activity of HO suppressor in plasma of the AD patients compared to healthy subjects is noticed. As discussed earlier, in the serum of AD patients, HO levels are increased which can be a significant diagnostic marker [13]. HO-1 protein levels were potentially raised in the hippocampus of AD subjects; in other words, HO-2 protein was potentially cut back in both AD and mild cognitive disorder of hippocampi [169]. Administering HO-1 is absolutely a neuroprotective response but it can show harmful effects in some incidences [27]. CO can also have a neuroprotective effect in case of treatment or destruction of the focal ischemia [68].

In a study of Schipper et al. (2009) it was shown that glial repression of HO-1 activity could be a potent therapeutic strategy for treatment of AD. One current study shows that, by selectively inhibiting of K_v2.1_ channel and promoting oxidant induced apoptosis, CO can work as a neuroprotective agent [170, 171].

Presently, it is shown that HO-1 or its metabolized product CO can reduce A*β*-induced toxicity in human neuroblastoma SH-SY5Y and in also rats of its primary hippocampal neurons which also involves modulation of K^+^ channels. Thus we can see that both HO-1 and CO protect cells from the toxicity of protofibrillar A*β*_1_, but this protection does not come from inhibition of apoptosis-associated K^+^ effects but rather comes from the inhibition of AMPK activation [172].

Thus we can see that there is debate between the beneficiary and detrimental effects of HO in case of AD patients.

#### 4.2.3. NO, Oxidative Stress, and AD

At very low concentrations, NO impedes cytochrome oxidase in competition with oxygen and it plays an essential role in regulation of physiology at the cellular energy metabolism. On the other hand, when the concentration is higher, the complexes from the respiratory chain are prevented by nitrosylation with the residues of tyrosine or protein thiol oxidation. When the concentration is very high, NO started to develop the formation of peroxynitrite anion (ONOO^−^), which is the product of reaction of NO plus superoxide; the resultant is the prevention of mitochondrial respiration and disturbance of many components of mitochondria through oxidative reactions. NO and its derivative named peroxynitrite cause halting of mitochondrial respiration in many organisms [173]. In AD and other neurodegenerative dementias, NO has a role in neurodegeneration and neuronal cell death by creating NO-mediated neurotoxicity [174]. *β*-Amyloid triggers microglial and astrocytic NO generation in AD [138, 175, 176]. Firstly, A*β* stimulates CD^4+^ T-cells and then activates microglial cells by producing cytokines [177]. iNOS activates NO release which is caused by the stimulated microglia. Activated microglia are generated by NO, the role of which is to mediate NO production [178]. When accretion of reactive astrocytes happens with the presence of cytokines, it directly imparts iNOS-mediated astrocytic NO production or has synergistic effects on astrocytic iNOS expression [175, 179]. In AD, iNOS activation and NO production are caused by neurofibrillary tangles (NFT); thus iNOS expression could be found in NFT-supporting neurons [180].

Some studies observed that partial deletion of dihydrolipoyl succinyltransferase enhances amyloid pathology and also oxidative stress as well as memory deficit in transgenic female mice model of AD [181]. Thus, a deficiency of manganese superoxide dismutase (MnSOD) raises deposition of amyloid [182], phosphorylation of tau [183], and also behavioral deficits [184]. In contrast, excessive expression of MnSOD causes reduced deposition of amyloid, oxidative stress, and also synaptic and memory deficits in two other transgenic mice models of AD [185, 186]. These results show that detoxification of the enzymes associated with the mitochondria has contribution to prevention of free radical accumulation as well as oxidative stress in AD [5]. Another report shows that neuroprotection is observed in transgenic AD mice after deletion of cyclophilin D which is a component of permeability of transition pore in mitochondria. CoQ10 is a coenzyme found within mitochondria also known as ubiquinone which is reported to have both antioxidant and neuroprotective properties both in vitro and in vivo [187] and shows great way in the treatment of neurodegenerative disorders [188].

Thus NO is responsible for causing oxidative stress interrupting mitochondrial respiration but its beneficiary effect of prevention and treatment of AD is notable.

## 5. Therapeutic Strategies Involving Neuronal Repair Especially in AD and PD by Modulating Oxidative Stresses

Studies with antioxidant which is shown in a transgenic mice model of AD that overexpresses APP and develops both of A*β* plaques and memory deficits. Experiment with an AD mice model with vitamin E supplementation in young but not aged mice is found to reduce A*β* plaques and deposition of amyloid [189]. Another experiment shows that curcumin, a polyphenol derived from curry spice, reduces damage by oxidation and amyloid pathology in Tg2376 AD mice model [190]. Another mice model showed phenolic compounds such as ferulic acid, myricetin, nordihyroguaianetic acid, and rosmarinic acid; administering those for 10 months and staining with 5 months of age in Tg2576 mice prevent the development of AD pathology by affecting aggregation of A*β* [189]. The ethanolic extract of the leaves of* Eriobotrya japonica* which is a traditional medicinal plant found in East Asia protects rat pheochromocytoma PC12 cells from A*β*^1–42^ induced cytotoxicity and also generation of ROS intracellularly [191]. Experiment with other synthetic compounds such as 2-Ethoxy-4, 5 –diphenol-1,3-oxazyme-6-one which has neuroprotective effects against H_2_O_2_ induced PC12 cell death [192]. Another compound named 5-chloro-7-iodo-quinolin-8-ol (clioquinol), which is recognized as iron chelator that blocks the generation of H_2_O_2_, induced toxicity by A*β*; thus it shows neuroprotective effect [189]. A compound named resveratrol, which is recognized as wine polyphenol, has effective neuroprotective feature in both in vitro and in vivo models of AD, PD, HD, and ischemic stroke and also in epilepsy [193]. A pesticide named rotenone which is naturally found in vegetable gardens that works as insecticide and when neurons are exposed to rotenone (0.3 *μ*M) which produce O^2^- that is inhibited by N-acetylcysteine. When human dopaminergic cells SH-SY5Y are treated with rotenone, the model is found to investigate new possible therapeutic targets for PD [194].

## 6. Conclusion

Gasotransmitters (H_2_S, CO, and NO) are the gaseous molecules which are produced and degraded naturally within the human body. From birth to death, they perform some specific functions in the body like formation of diseases and mediator of physiologic conditions and sometimes they act as a reinforce to protect body from the oxidative stresses such as reactive oxygen species (ROS) and reactive nitrogen species (RNS), which are the main reasons or causes of neuroinflammation as well as neurodegenerative diseases such as Alzheimer's disease (AD) and Parkinson's disease (PD). Gasotransmitters such as H_2_S, CO, and NO have different protective effects against oxidative stress and a contribution in mediating neurodegenerative conditions as well as neural repair but those can be neurotoxic when they are excessively produced by the body. All of these gasotransmitters have both neuroprotective and neurodegenerative effects in the body which we have learned from various rodent models, although the mechanisms they work on it to mediate body homeostasis is still unknown. Further research on these gasotransmitters and knowing their various mechanisms and pathways can open a door to find new pharmaceuticals to treat neurodegenerative diseases.
